# Potential mechanisms underlying the effect of walking exercise on cancer-related fatigue in cancer survivors

**DOI:** 10.1007/s11764-024-01537-y

**Published:** 2024-01-31

**Authors:** Isa Hiske Mast, Coen C. W. G. Bongers, Elske C. Gootjes, Johannes H. W. de Wilt, Maria T. E. Hopman, Laurien M. Buffart

**Affiliations:** 1 Department of Medical BioSciences, Radboud University Medical Center, P.O. Box 9101, 6500 HB Nijmegen, the Netherlands; 2 School of Sport and Exercise, HAN University of Applied Sciences, Nijmegen, the Netherlands; 3 Department of Medical Oncology, Radboud University Medical Center, Nijmegen, the Netherlands; 4 Department of Oncological Surgery, Radboud University Medical Center, Nijmegen, the Netherlands

**Keywords:** Cancer-related fatigue, Exercise intervention, Muscle contractile properties, Resilience

## Abstract

**Purpose:**

Cancer-related fatigue (CRF) is a common and debilitating long-term side effect of cancer and its treatment. While exercise has been shown to effectively reduce CRF, the underlying mechanisms are not fully clear. Therefore, the aim of this study was to explore the effects of a 4-month walking exercise program on fatigue severity and to explore potential underlying physiological, behavioral, and psychological mechanisms of action.

**Methods:**

We included 27 cancer survivors (59 ± 15 years, 37% female) with variable cancer diagnoses who were at least moderately fatigued and finished treatment between 6 and 36 months ago. This study with a quasi-experimental interrupted time-series design compared a 4-month walking intervention period with a 4-month control period. Measurements of fatigue and physiological, behavioral, and psychological factors were performed, supplemented with participants’ perceptions on how exercise influenced their fatigue.

**Results:**

A significant and clinically relevant decrease in fatigue severity was found over time (β =  − 8.1, 95% CI =  − 12.1; − 4.2), but could not be attributed directly to the walking exercise intervention. Increases in muscle strength (β =  − 0.07, 95% CI =  − 0.12; − 0.02), physical activity (β =  − 0.1, 95% CI =  − 0.2; − 0.04), and sleep quality (β = 1.1, 95% CI = 0.3; 1.9), as well as decreases in muscle relaxation times (β = 0.09, 95% CI = 0.02; 0.16) and psychological distress (β = 1.1, 95% CI = 0.8; 1.3) were associated with reductions in fatigue severity. Resilience and physical well-being were perceived as most important constructs explaining the walking exercise effects on fatigue.

**Conclusion:**

Our findings reveal potential physiological, behavioral, and psychological mechanisms underlying the multidimensional effects of exercise on fatigue severity.

**Implications for Cancer Survivors.:**

Incorporating resistance exercise and addressing resilience and physical well-being might improve the efficacy of exercise interventions for cancer survivors.

**Supplementary Information:**

The online version contains supplementary material available at 10.1007/s11764-024-01537-y.

## Introduction

Cancer-related fatigue (CRF) is one of the most prevalent side effects of cancer and its different treatment modalities [1]. It is defined as a distressing, persistent subjective sense of physical, emotional, and/or cognitive tiredness or exhaustion related to cancer or cancer treatment that is not proportional to recent activities and interferes with usual functioning [2]. Fatigue mostly occurs during active cancer treatment, however, one out of four cancer survivors report CRF after completion of treatment, severely impacting quality of life (QoL) [3, 4].

The pathogenesis of CRF is multifactorial and poorly understood. Risk factors such as type of cancer, treatment regimens, and patient characteristics can contribute to CRF [3, 5]. As such, behavioral and psychological factors including physical (in)activity, sleep quality, anxiety, and depression have been associated to CRF [6]. Additionally, proposed pathophysiological mechanisms contributing to CRF include inflammation, reduced aerobic fitness, reduced muscle strength, circadian rhythm disruption, altered heart rate variability, and impaired neuromuscular function [7, 8].

Peripheral muscular fatigue may be an important neuromuscular mechanism related to CRF [7, 8]. It is defined as the loss of voluntary force-producing capacity during exercise, and can be determined using electrical stimulations [9]. However, muscle fatigue can also be of central origin caused by failure of neural drive from the central nervous system (CNS) leading to a loss in voluntary muscle force production [8, 9]. Investigating muscle contractile properties could provide a better understanding of muscular changes in patients with CRF, thereby gaining insight in potential pathophysiological mechanisms contributing to CRF. At present, the contribution of various physiological, behavioral, and psychological factors to CRF remain to be elucidated [7].

Exercise has been identified and recommended as an effective non-pharmacological treatment of CRF [6, 10]. Meta-analyses indicate a small-to-moderate effect from exercise interventions on CRF with a large heterogeneity in response [11]. A more thorough understanding of this heterogeneity can be achieved by identifying the underlying mechanisms of exercise effects on CRF. This knowledge will help to further personalize interventions to improve their effects.

Exercise can affect CRF via physiological, behavioral, and psychological pathways. Physiological pathways include improved aerobic fitness, motor neuron firing rate, maximal voluntary muscle activation, and released anti-inflammatory cytokines [12–14]. Behavioral pathways include a higher physical activity level and improved sleep quality [11, 15]. Psychological pathways include reduced symptoms of anxiety and depression [16]. Despite the positive effects of exercise on CRF, fatigue has also been identified as a barrier to exercise [17]. Walking exercise is a moderate-intensity exercise type with limited health risks and has been indicated by cancer survivors as their preferred exercise type [18], reducing the barrier to exercise.

**Fig. 1:**
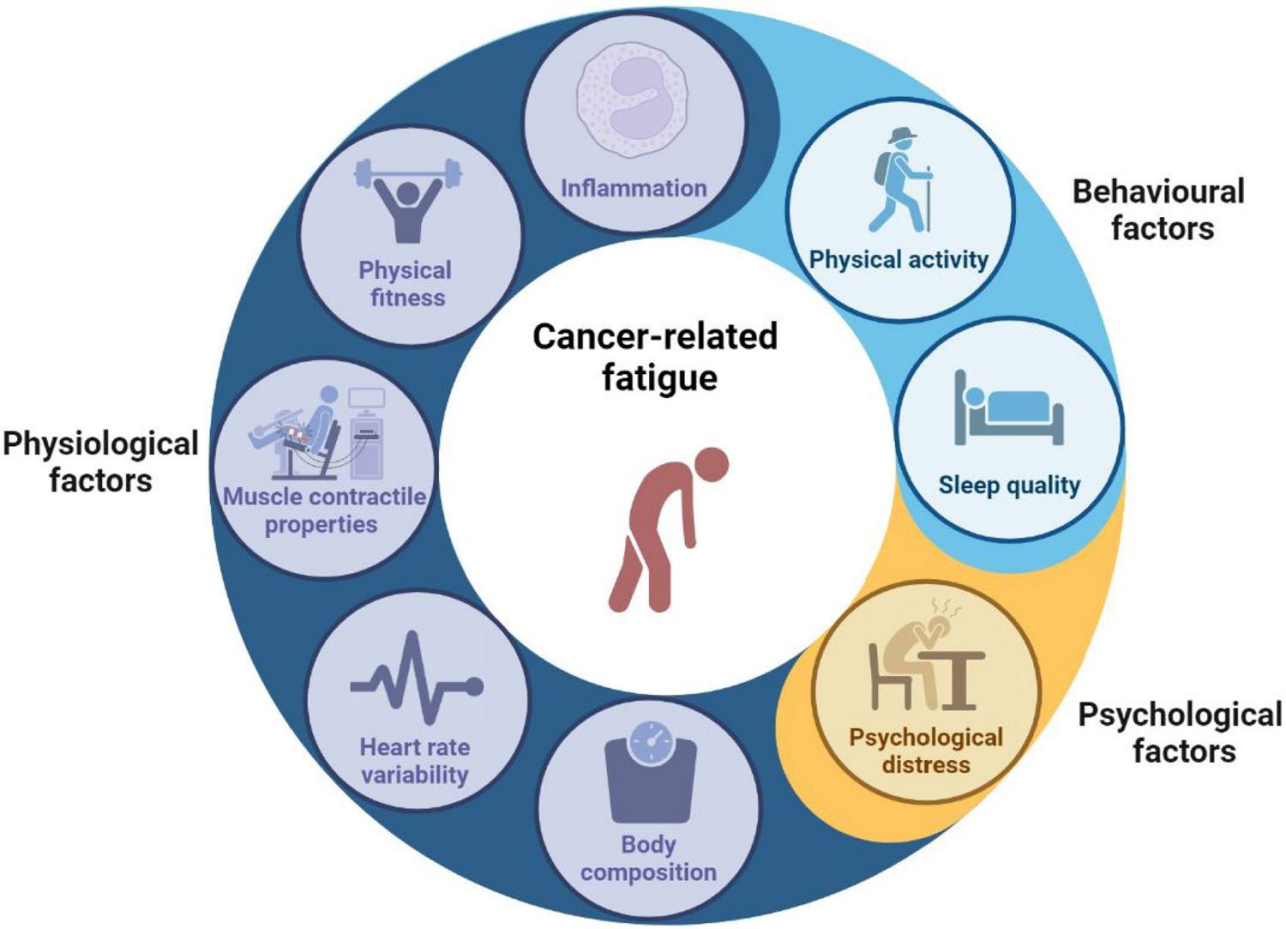
Potential physiological, behavioral, and psychological mechanisms underlying cancer-related fatigue investigated in the KINETICS trial

Therefore, we launched the walking exercise training to reduce fatigue in cancer survivors (KINETICS) trial to examine which physiological, behavioral, and psychological pathways can explain the effects of a 4-month walking exercise program on self-reported CRF (Fig. 1). To allow for a comprehensive assessment of all potential pathways, we also captured cancer survivors’ perceptions of how a walking exercise intervention influenced their CRF.

## Methods

### Participants

This explorative study aimed to include 40 cancer survivors with self-reported CRF. They were recruited by advertisements distributed by patient organizations, by local and national media, and via the Department of Surgery of Radboud University Medical Center. Subsequently, they were screened for eligibility via telephone contact. To be eligible for inclusion, cancer survivors needed to be at least moderately fatigued, as indicated with a fatigue score above 27 on the subscale fatigue of the checklist individual strength (CIS; [19]) at the time of screening, and aged 18 years or older. Additionally, oncological treatment needed to be completed between 6 months and 3 years ago, except for hormone therapy, and cancer survivors needed to be in a long-term stable clinical situation. Cancer survivors were not eligible if they had neurological or orthopedic health problems hampering walking exercise, had hemoglobin levels below 6.0 mmol/l, or glucose levels above 8.0 mmol/l at the time of inclusion. This study was approved by the regional ethical review board (METC Oost-Nederland; #2019–6065), and all participants gave written informed consent before enrollment.

### Design and intervention

The study has a quasi-experimental interrupted time-series design, in which participants act as their own controls. All study measurements took place at three time points: baseline (T0), pre-intervention (T1), and post-intervention (T2). The 4-month period between baseline and the pre-intervention visit had no study-related activities and served as a control period. After the T1 measurement, cancer survivors received a 4-month home-based walking exercise program. The program was individually tailored and included a detailed description of weekly training goals that gradually increased in training duration, frequency, and intensity leading towards a personal walking exercise goal. Three sessions of moderate intensity walking exercise with an individually tailored duration that progressed over time were prescribed per week. Additionally, one weekly session of home-based resistance training consisting of six body weight exercises was added to the walking program. Cancer survivors received exercise counselling by phone or email every other week from the research coordinator to monitor the training program and adjust the program if necessary. During the counselling sessions behavior change techniques including, among others, goal setting, action planning, and problem-solving, were applied to support motivation and program adherence [20] (Supplementary material 1).

### Cancer-related fatigue

Fatigue severity was assessed using the fatigue subscale of the Checklist Individual Strength (CIS), which consists of 8 items scored on a 7-point Likert scale [19]. Scores can be divided in the categories: normal fatigue (score 8–26), moderate fatigue (score 27–34), and severe fatigue (score 35–56). The CIS is a validated questionnaire and has extensively been used in cancer survivors [21, 22]. Minimally clinically important difference (MCID) of the fatigue subscale is 8 points [23]. Additionally, the Multidimensional Fatigue Inventory (MFI) was included as secondary outcome to evaluate physical and mental fatigue dimensions. MFI scores range between 4 and 20 [24]. In addition, vitality was assessed using the RAND-36 health survey (RAND-36), in which raw scores are linearly converted to a 0 and 100 score, and higher scores representing higher vitality [25].

### Physiological factors

Height (m), weight (kg), and body composition (skeletal muscle mass and body fat mass in kilograms) were obtained (InBody 770, Biospace, Seoul, Republic of Korea). Aerobic fitness (maximum oxygen uptake; VO_2_max) was estimated using the Ästrand-Rhyming submaximal exercise test on an cycle ergometer (Lode Corival; Lode, Groningen, the Netherlands; [26]). The 1 repetition maximum (1RM) based on the indirect 1 repetition maximum test on a leg press (EN-Dynamic, Enraf–Nonius, Rotterdam, the Netherlands; [27]) was used as indicator for lower body muscle strength. Heart rate variability (HRV) was measured in supine position after 10 min of relaxation in supine position. During the HRV measurements, consecutive R-R peak intervals were recorded for 5 min (Polar V800, Polar Electro Oy, Kempele, Finland), while breathing frequency was paced by a metronome at a frequency of 12 breaths per minute. R-R intervals were analyzed using Kubios (version 3.5, Biosignal Analysis and Medical Imaging Group, Kuopio, Finland) to determine time domain (SDNN; standard deviation of N–N intervals, RMSSD; root mean square of successive RR interval differences) and frequency domain variables (LF/HF ratio; low frequency/high frequency ratio) [28].

Muscle contractile properties of the dominant *Quadriceps femoris* muscle were determined using an electrical stimulation protocol [29] and included maximal voluntary contraction (MVC), muscle fatiguability, early- and half relaxation time (Rt), and maximal force rise (MFR). To evaluate muscle contractile properties, participants were seated in upright position. The lower leg was fixated to a force transducer, and surface electrodes were placed on the distal and proximal part of the anterior thigh [29, 30]. MVC was determined by instructing the participants to maximally extend the knee for at least 3 s and calculating the mean maximal force over a stable interval of approximately 1 s. Subsequently, the muscle was electrically stimulated inducing a force of least 40% of the MVC. Muscle fatiguability was assessed by repetitively stimulating the quadriceps muscle for 2 min using 30-Hz bursts with a duration of 1 s every 2 s [30].

Force signals were analyzed using Matlab (Version R2022a; The MathWorks Inc., Natick, Massachusetts). Muscle fatiguability was evaluated by calculating peak force decline using the percentage force decline between the first and last three bursts of the fatiguability protocol. Early and half relaxation time were calculated, defined as the time needed for the force to decline from 75 to 50% and from 50 to 25% of peak force, respectively. Maximal force rise (MFR) was calculated as the percentage of maximal force incline divided by the peak force [29].

Venous blood was collected and stored in a − 80 °C freezer. At the end of the study, serum samples were analyzed using an immuno-oncology Luminex assay (Assay HCYTA-60 K, Merck-Millipore, Burlington, USA). This panel measures inflammatory markers interleukin 6 (IL-6) and tumor necrosis factor α (TNF-α). Blood plasma samples were analyzed using an immunoturbidimetric assay (Roche diagnostics, Indianapolis, USA) to quantitatively evaluate C-reactive protein (CRP) concentration. Values below limit of quantification were approximated as 75% of the lowest level of quantification, and values below limit of detection were assigned a placeholder value of 0.01 mg/l for CRP, 0.01 pg/ml for IL-6, and 0.1 pg/ml for TNF-α.

### Behavioral and psychological factors

Physical activity was measured objectively using an accelerometer (ActivPAL micro, PAL technologies, Glasgow, UK). The accelerometer was placed on the upper thigh and worn 24 h a day for at least 7 continuous days. Raw data was converted using PAL Analysis software (PAL Software Suite, version 8, PAL Technologies), analyzed using a script adapted from Winkler et al. [31], and subsequently divided in moderate-to-vigorous physical activity (MVPA; metabolic equivalent (MET) values ≥ 3) and sedentary behavior (MET values ≤ 1.5).

The Short Questionnaire to Assess Health-Enhancing Physical Activity (SQUASH; [32]) was used to evaluate self-reported physical activity. The amount of moderate-to-vigorous physical activity during leisure time and the total amount of activity during the week were evaluated and converted to MET values based on the updated compendium of Ainsworth [33].

Sleep quality was assessed using the Pittsburgh Sleep Quality Index (PSQI). The PSQI can be divided into seven components, which can be summed to a global sleep quality score ranging from 0 to 21 [34].

Symptoms of psychological distress were assessed using the Hospital Anxiety and Depression Scale (HADS), which yields a total score ranging from 0 to 42, with higher scores indicating higher distress [35].

### Covariables

Sociodemographic information, clinical information, and smoking behavior were assessed using a custom-made questionnaire. Categorical variables included marital status (married, divorced, living together, widow), education level (low, medium, high), treatment type and number of treatments (surgery, radiotherapy, chemotherapy, hormone therapy, immunotherapy), and smoking status (current, former, never). Comorbidities were assessed using a self-reported version of the Charlson Comorbidity Index (CCI; [36]).

### Cancer survivors’ perspectives

Cancer survivors’ perspectives on how walking exercise influenced their CRF were captured using concept mapping [37]. Experiences were collected in response to the focus statement “*How did walking exercise influence your perceived fatigue?*”. Four 1-h brainstorm sessions were organized in small groups (4–6 participants) during the post-intervention study visit, after completion of the intervention and study measurements. During the brainstorm sessions, cancer survivors were asked to write down all their experiences in response to the focus statement. Subsequently, cancer survivors were asked to share their experiences and discuss whether the experiences were defined accurately and unambiguous. After collecting all experiences, researchers removed identical experiences and created a list of all individual experiences. Negatively framed statements were not included in this analysis to prevent indistinct clusters. Following the brainstorm sessions cancer survivors clustered all individual experiences in minimally three clusters of at least two experiences using an online tool (HvA Concept Mapping Tool; Hogeschool van Amsterdam, the Netherlands), and provided all clusters with a corresponding title. Additionally, they rated all individual experiences on a 5-point Likert scale from “unimportant” to “very important.” Concept maps were created in R studio (Version 2022.02.1, R Core Team (2022)) using R-CMAP, an open-source software for concept mapping [38]. This was done by transforming the individual patient data through a multidimensional scaling algorithm, whereafter a 2-dimensional representation of the relative distances between statements was provided. Based on these relative distances, clusters were formed by hierarchical clustering using Ward’s method [38].

### Statistical analyses

Demographical and clinical characteristics of cancer survivors were summarized by means and standard deviations (SDs) or medians and interquartile ranges for continuous variables, as appropriate, and numbers and proportions for nominal variables. Linear mixed models were used to examine the change in fatigue severity over time. Subsequently, physiological, behavioral, and psychological variables were added to the model (separately for each variable) to examine whether changes in these variables were associated with changes in fatigue severity. Assumptions for linearity, normality of residuals, and homoscedasticity were met for all models and models were adjusted for sex. All analyses were conducted in R studio (Version 2022.02.1, R Core Team (2022)).

## Results

Between June 2021 and October 2021, 57 cancer survivors responded to the advertisements and invitations and were subsequently screened for participation. In total, 30 cancer survivors were not eligible due to low fatigue scores (*n* = 10), an inadequate time period after treatment completion (*n* = 7), lack of interest (*n* = 7), already participating in regular physical exercise (*n* = 3), or unknown reasons (*n* = 3). Consequently, 27 cancer survivors were included in the study. Four participants dropped out from the study during the control period, and two participants dropped out during the intervention period. Reasons for dropout were physical complaints hampering walking exercise (*n* = 5) or disease recurrence (*n* = 1). Cancer survivors were on average 59 ± 15 years old, and 37% were female (Table 1).

**Table 1:** Participant characteristics

	Participants (*n* = 27)
Age, mean ± SD (years)	59 ± 15
Gender, *n* (%) female	10 (37.0)
BMI, mean ± SD (kg/m^2^)	26.9 ± 5.0
Marital status, *n* (%)	
Married	18 (66.7)
Divorced	3 (11.1)
Living together	4 (14.8)
Widow	2 (7.4)
Education level, *n* (%)	
Low	4 (14.8)
Middle	13 (48.1)
High	10 (37.0)
Smoking status, *n* (%)	
Current	2 (7.4)
Former	12 (44.4)
Never	10 (37.0)
Unknown	3 (11.1)
Cancer type, *n* (%)	
Gastrointestinal cancer	9 (33.3)
Gynecological cancer	6 (22.2)
Urogenital cancer	4 (14.8)
Hematological cancer	3 (11.1)
Breast cancer	2 (7.4)
Lung cancer	1 (3.7)
Brain tumor	1 (3.7)
Melanoma	1 (3.7)
Treatment type, *n* (%)*	
Surgery	18 (66.7)
Radiotherapy	14 (51.9)
Chemotherapy	18 (66.7)
Hormone therapy	5 (18.5)
Immunotherapy	3 (11.1)
Number of treatments, median [IQR]	2 [2-3]
Current hormone therapy, *n* (%)	5 (18.5)
Self-reported CCI, median [IQR]	3 [2-4]
Polypharmacy (> 2 medications), *n* (%)	4 (14.8)
Time between treatment completion and study inclusion (years), median [IQR]	1.5 [1-2]

*Participants may have received multiple treatment types. Abbreviations: *BMI* body mass index, *CCI* Charlson comorbidity index

### Fatigue

During the total study period, perceived fatigue severity (β =  − 8.1, 95% CI =  − 12.1; − 4.2) and physical fatigue (β =  − 1.7, 95% CI =  − 3.1; − 0.3) decreased, and vitality increased (β = 8.5, 95% CI = 2.8; 14.2; Table 2). Fatigue severity decreased significantly during the control period (β =  − 5.9, 95% CI =  − 9.7; − 2.1), and showed no significant changes during the intervention period (Table 2). During the control period, also mental fatigue decreased (β =  − 1.5, 95% CI =  − 2.8; − 0.2), and vitality increased (β = 8.8, 95% CI = 3.2; 14.3; Table 2), while no significant changes in these outcomes were found during the intervention period.

**Table 2:** Descriptive information and longitudinal changes in dimensions of fatigue

	**T0**	**T1**	**T2**	**Total study period**	**Control period**	**Intervention period**
				*β [95% CI]*	*β [95% CI]*	*β [95% CI]*
Fatigue severity	35 ± 9	28 ± 13	25 ± 12	− 8.1 [− 12.1; − 4.2]*	− 5.9 [− 9.7; − 2.1]*	− 2.2 [− 6.2; 1.7]
Mental fatigue	12 ± 5	11 ± 4	11 ± 4	− 0.9 [− 2.2; 0.5]	− 1.5 [− 2.8; − 0.2]*	0.6 [− 0.8; 2]
Physical fatigue	12 ± 4	11 ± 4	10 ± 4	− 1.7 [− 3.1; − 0.3]*	− 0.8 [− 2.2; 0.5]	− 0.8 [− 2.2; 0.6]
Vitality	56 [47–62]	62 [50–75]	62 [56–69]	8.5 [2.8; 14.2]*	8.8 [3.2; 14.3]*	− 0.2 [− 6; 5.5]

Variables are displayed as mean ± SD or median (IQR). Regression coefficients (β) and 95% confidence intervals (CI) represent the mean change in the variable over time assessed using unadjusted linear mixed models. Higher scores on the subscale fatigue severity (range 20–140), mental fatigue (range 4–20), and physical fatigue (range 4–20) indicate more fatigue. Higher scores on the subscale vitality (range 0–100) represent higher vitality. * = *p* value < 0.05

### Physiological factors

Descriptive information and changes over time for all physiological factors can be found in Supplementary material 2. During the control period, muscle strength and IL-6 concentration increased significantly. During the intervention period, VO_2_max, early relaxation time, and TNF-α concentration increased significantly. During the total study period, VO_2_max, early relaxation time, and TNF-α increased significantly, and the decrease in maximal force rise during the fatiguability electrical stimulation protocol reduced.

**Fig. 2:**
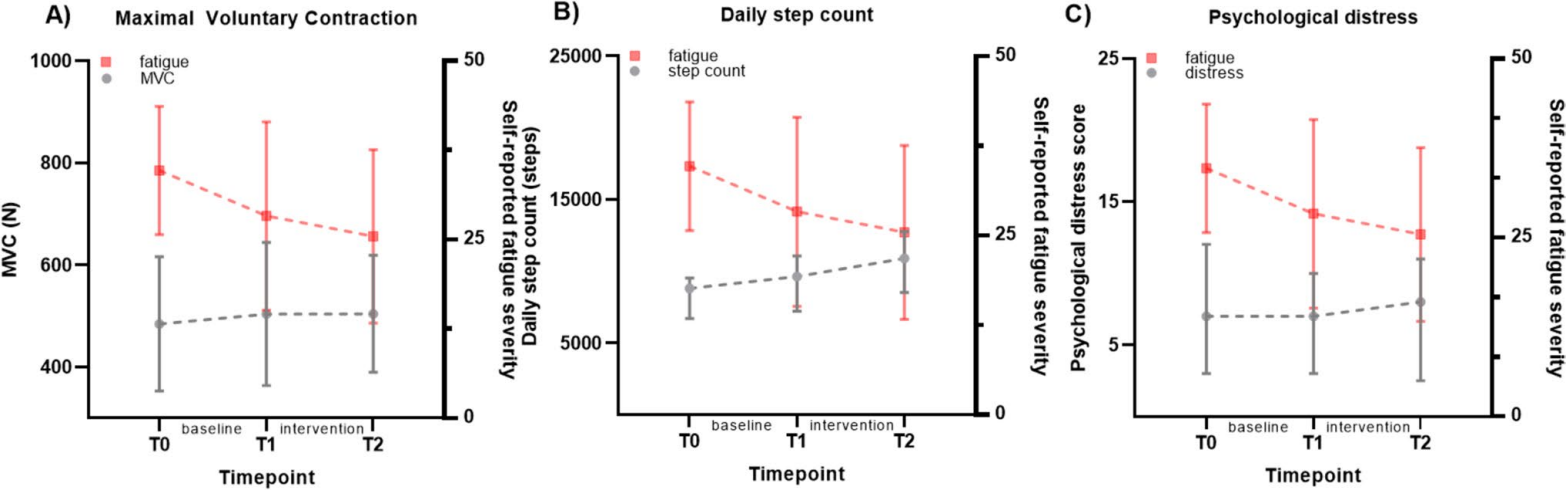
Longitudinal associations with changes in fatigue severity for **A** maximal voluntary contraction (MVC) of the quadriceps femoris muscle, **B** daily step count, and **C** psychological distress. Values are presented as mean ± standard deviation for fatigue severity and MVC and median (IQR) for daily step count and psychological distress

**Fig. 3:**
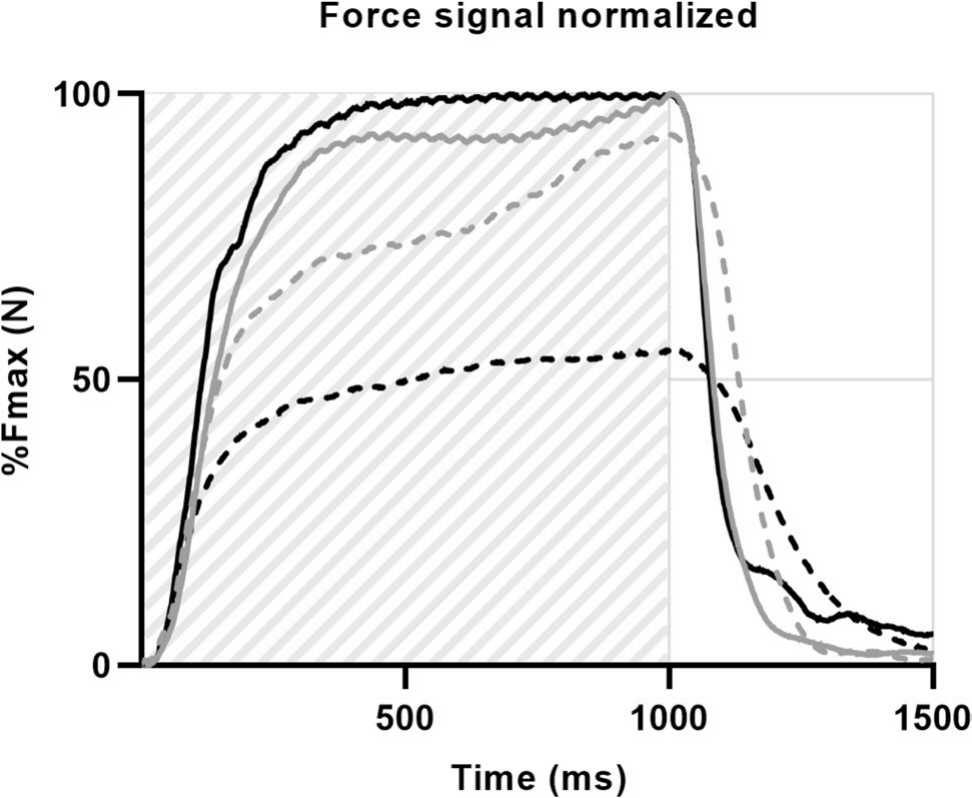
Representative force signals of the first (straight) and last (dashed) electrically stimulated muscle contractions of the fatiguability protocol from a severely fatigued (black) and less fatigued (gray) person. The muscle is stimulated for 1 s (striped light grey) and relaxes directly after the electrical pulse

Increases in muscle strength (per 1RM in kg, β =  − 0.07, 95% CI =  − 0.12; − 0.02; per 1RM in kg/kg body weight, β =  − 5.1, 95% CI =  − 9.4; − 0.8), maximal voluntary contraction (per MVC in N: β =  − 0.04, 95% CI =  − 0.06; − 0.01; Fig. 2), and reductions in muscle early relaxation time during the fatiguability protocol (per %: β = 0.09, 95% CI = 0.02; 0.16) and muscle half relaxation time (per %: β = 0.07, 95% CI = 0.01; 0.14) were significantly associated with a decrease in fatigue severity (Fig. 3, Table 3). Changes in aerobic fitness, body composition, heart rate variability, and concentrations of CRP, TNF-a and IL-6 were not significantly associated with changes in fatigue severity.

**Table 3:** Longitudinal associations of physiological, behavioral, and psychological factors with fatigue severity

	β (95% CI) total study period	β (95% CI) total study period^†^
Physiological		
Body composition		
BMI (kg/m^2^)	− 0.3 (− 1; 0.4)	
Skeletal muscle mass (kg)	− 0.8 (− 1.7; 0.1)	
Body fat mass (kg)	− 0.1 (− 0.4; 0.2)	
Physical fitness		
Estimated VO_2_max	− 0.2 (− 0.5; 0.1)	
Estimated 1RM (kg)	− 0.07 (− 0.12; − 0.02)*	
Estimated 1RM (kg/kg body weight)	− 5.1 (− 9.4; − 0.8)*	
MVC (N)	− 0.04 (− 0.06; − 0.01)*	
MVC (N/kg body weight)	− 1.7 (− 3.5; 0.2)	
Muscle contractile properties		
Muscle fatiguability (%)	− 0.2 (− 0.5; 0.1)	
Early relaxation time (ms)	0.05 (− 0.89; 0.99)	
Increase early relaxation time (%)	0.09 (0.02; 0.16)*	
Half relaxation time (ms)	0.04 (− 0.39; 0.45)	
Increase half relaxation time (ms)	0.07 (0.01; 0.14)*	
Maximal force rise (%/ms)	11.0 (− 4.3; 25.8)	
Decrease Maximal force rise (%)	− 0.08 (− 0.21; 0.05)	
Heart rate variability		
SDNN	0.00 (− 0.05; 0.05)	
RMSSD	− 0.01 (− 0.06; 0.03)	
LF/HF ratio	0.05 (− 0.33; 0.41)	
Inflammation		
TNF-α (pg/ml)^†^	0.03 (− 1.27; 1.28)	1.03 (0.28; 3.60)
IL-6 (pg/ml)^†^	0.72 (− 0.34; 1.78)	2.05 (0.71; 5.93)
CRP (mg/l)^†^	0.61 (− 0.24; 1.44)	1.84 (0.79; 4.22)
Behavioral		
Objectively assessed behavior		
Daily step count (per 100 steps)	− 0.11 (− 0.17; − 0.04)*	
MVPA (hours/week)	− 1.21 (− 1.95; − 0.48)*	
Sitting time (hours/day)	0.7 (− 0.8; 2.2)	
Self-reported behavior		
MVPA leisure time (hours/week)	− 0.2 (− 0.49; 0.09)	
MET hours/week^†^	− 3.1 (− 7.1; 0.77)	0.04 (0.00; 2.16)
Sleep quality		
Total sleep score (PSQI)	1.1 (0.3; 1.9)*	
Psychological		
Distress	1.1 (0.8; 1.3)*	

Regression coefficients* (*β) with corresponding 95% confidence intervals (CIs) represent the association between the variable and fatigue over time (averaged over all time points), adjusted for sex. Abbreviations: *BMI* body mass index, *VO*_*2*_*max* maximum oxygen uptake, *1RM* 1 repetition maximum; *SDNN* standard deviation of N–N intervals, *RMSSD* root mean square of successive RR interval differences, *LF/HF* ratio low-frequency/high-frequency ratio, *MVPA* moderate-to-vigorous physical activity, *MET* metabolic equivalent of task. ^†^Values of these variables are log-transformed and are presented in this column after back transformation to original scale, * = *p* value < 0.05.

### Behavioral and psychological factors

Objectively measured daily step count and weekly MVPA increased during the total study period (Supplementary material 2). Increases in objectively measured daily step count (per 100 steps: β =  − 0.1, 95% CI =  − 0.2; − 0.04), weekly MVPA (per hour: β =  − 1.21, 95% CI =  − 1.95; − 0.48), sleep quality (β = 1.1, 95%CI = 0.3; 1.9), and decreases in psychological distress (β = 1.1, 95% CI = 0.8; 1.3) were significantly associated with decreases in fatigue severity (Table 3; Fig. 2).

### Cancer survivors’ perspectives

**Fig. 4:**
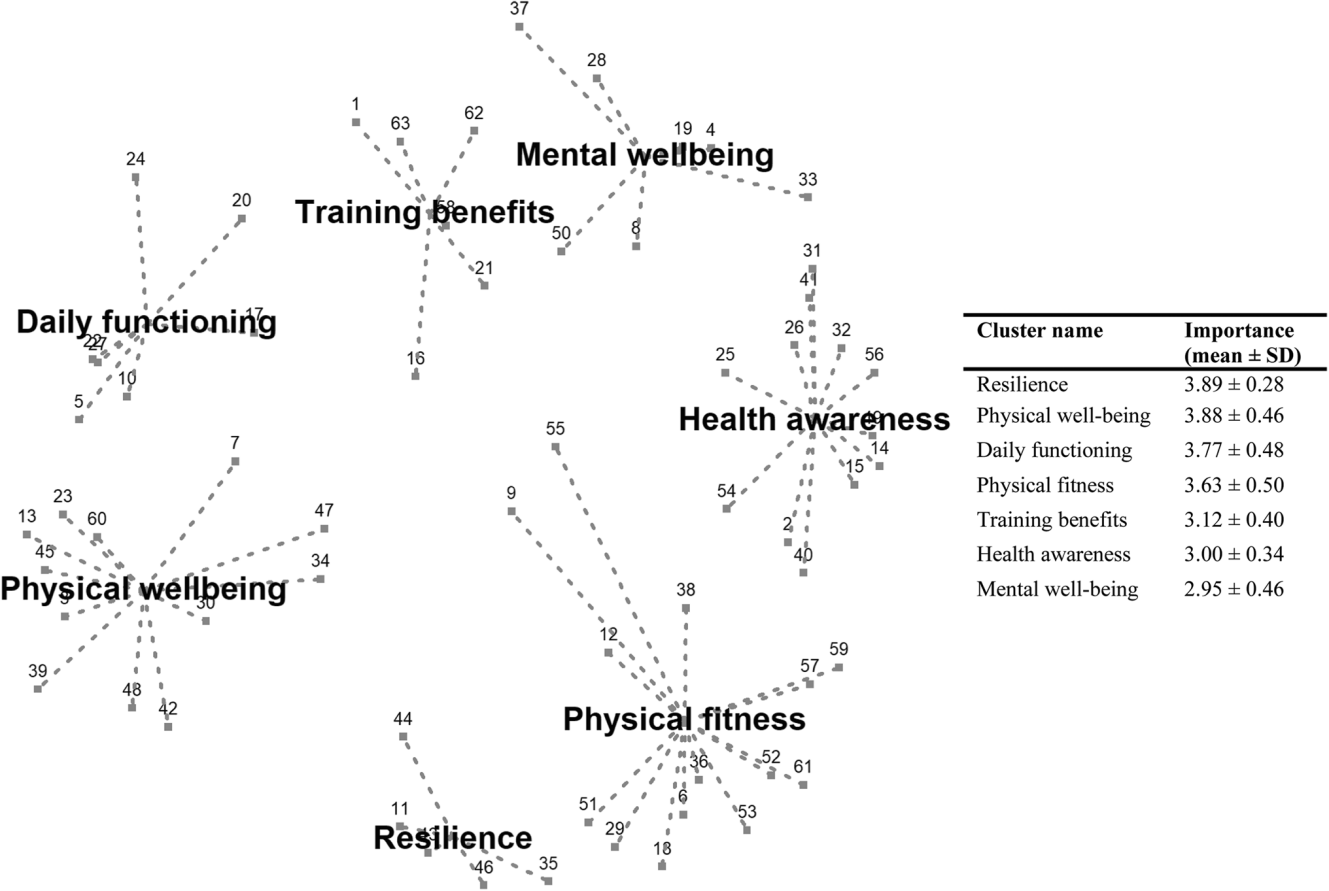
Concept map of patients’ experiences of how walking exercise reduces their fatigue severity. Each point corresponds to a statement patients formulated in response to the focus statement (see Supplementary material 3). Statements that are frequently placed in the same cluster are presented closer together in this figure. The dotted lines help to illustrate relative distances. Cluster names of each cluster are presented in the figure. The accompanying table displays the cluster names, as well as the mean and standard deviation of their respective importance scores

In total, 19 cancer survivors generated 111 statements. Removal of 29 duplicates and 19 statements that were not framed positively, resulted in 63 positively framed statements. The 63 statements were clustered in seven clusters, and presented in a concept map (Fig. 4). The seven clusters included *training benefits*, *mental well-being*, *health awareness*, *physical fitness*, *resilience*, *physical well-being*, and *daily functioning*. The cluster *resilience* was rated as the most imported cluster with a mean importance of all statements in the cluster of 3.89 ± 0.28, followed by *physical well-being* (3.88 ± 0.46), and *daily functioning* (3.77 ± 0.48). A description of the individual statements and their importance are provided in Supplementary material 3.

## Discussion

This study investigated the effect of a 4-month walking intervention on cancer-related fatigue and studied physiological, behavioral, and psychological variables associated with these effects. Our results showed that cancer survivors showed a clinically relevant decrease in fatigue severity, however, this effect already started before the walking intervention. Additionally, increases in muscle strength, physical activity, sleep quality, and reductions in muscle relaxation times and psychological distress were associated with reductions in fatigue severity. Furthermore, cancer survivors perceived that improved resilience and physical well-being were the most important benefits of the walking intervention that contributed to reduced perceived fatigue. 

### Cancer-related fatigue

The present study showed a clinically relevant decrease in fatigue severity over time to below the cut-off value for moderate fatigue [23]. Unexpectedly, the largest change was already found in the control period. Since sudden natural recovery is unlikely in cancer survivors with long-term fatigue, we hypothesize that cancer survivors already started with their walking exercise prior to the start of the intervention period, thereby introducing contamination. This hypothesis is supported by the average increase in step count and MVPA of approximately 10% during the control period. Hence, explaining and participating in the study may already have impacted exercise behavior [39], leaving less room for further changes during the intervention period.

### Underlying mechanisms

Our finding that changes in muscle contractile properties (specifically the increase in relaxation times during a fatiguability protocol) were associated with reductions in fatigue severity complements results from previous studies showing that increases in rate of force development was correlated with reductions in perceived fatigue in patients with breast or colon cancer undergoing chemotherapy [40] and that decreases in half relaxation time after a fatiguability electrical stimulation protocol was associated with decreases in fatigue in patients with chronic myeloid leukemia [41]. As skeletal muscle relaxation times are mostly related to Ca^2+^ reuptake into the sarcoplasmic reticulum [42], the increases in relaxation times may suggest sarcoplasmic reticulum dysfunction in cancer survivors with CRF. Additionally, cytostatic agents used in oncological treatment can disrupt muscle relaxation and calcium homeostasis [43] and induce mitochondrial dysfunction. Mitochondrial dysfunction can result in an impaired adenosine triphosphate (ATP) generating capacity [44] which impairs the active reuptake of calcium necessary for muscle relaxation [43]. Physical exercise (preferably high intensity interval endurance exercise or resistance exercise [45]) during and after cancer treatment can increase mitochondrial density and function and thereby reducing fatigue [46, 47]. However, future studies should reveal whether exercise may improve sarcoplasmic reticulum function in cancer survivors and thereby reduce fatigue.

Next to muscle relaxation times, we found that a higher quadriceps muscle strength was associated with lower fatigue severity and that cancer survivors recognized improved fitness and muscle strength as a mechanism through which exercise helped to reduce fatigue. Strikingly, muscle strength from cancer survivors in our study was approximately 30% lower, also after the intervention, compared to values in middle-aged healthy individuals that were assessed using the same protocol [30, 48], and were comparable with patients after chemotherapy treatment [40]. The low values of muscle strength and the association with fatigue, suggest that improving muscle strength after treatment could be important to prevent long-term fatigue. On the contrary, neither the increase in aerobic fitness during the intervention period, nor changes in body composition, and heart rate variability were associated with fatigue severity, which indicate that these variables may be a less important intervention target to reduce fatigue. While previous studies have found no differential effect on fatigue across exercise programs [11], results from this study suggest to include resistance exercises to improve muscle strength combined with progressive muscle relaxation training to improve muscle relaxation times [49]. A multimodal exercise intervention including improving physical activity, resistance exercise, and progressive muscle relaxation may therefore be a promising approach to enhance efficacy of exercise interventions in cancer survivors.

Results of our study also identified several behavioral and psychological pathways via which exercise can reduce fatigue severity, including improved physical activity and sleep and reduced distress. Our findings demonstrate that an increase in physical activity and sleep quality is associated with reductions in fatigue severity, supporting international physical activity guidelines for cancer survivors [10] and previous literature [11, 15]. Also, cancer survivors in our study perceived that exercise helped them to sleep better and improved their well-being, thereby reducing fatigue. Augmenting sleep quality can help maintain a steady circadian rhythm, which is also associated with a reduction in CRF [7, 50]. The associations between reduced psychological distress and reduced fatigue severity support results from a previous meta-analysis showing that symptoms of anxiety and depression are associated with fatigue [3, 16], and can be reduced by exercise [10, 51]. Resemblance of symptoms of psychological distress and fatigue make it difficult to speculate about causality of this association [16], however, exercise clearly benefits both symptoms [10].

Additionally, cancer survivors identified that improved physical and mental resilience (i.e., process of adaptation in response to threats or adversity [52]) and physical well-being were most important mechanisms explaining reductions in fatigue severity after walking exercise. This finding supports a previous cross-sectional study among patients with cancer showing that higher physically activity levels are associated with better resilience [53]. Additionally, patients with cancer perceived that exercise provided a means to contribute themselves to recovery and to improve coping strategies, such as maintaining a positive attitude [54]. In our intervention, we incorporated several behavior change techniques, including enhancement of self-efficacy, active coping, and social support, which have shown to be important resilience promoting strategies [55]. The hypothesis that improved resilience and physical well-being mediate the effects of exercise on cancer-related fatigue should be confirmed in future trials.

### Strengths and limitations

The strength of this study is its multifactorial approach, incorporating physiological, behavioral, and psychological factors including detailed measurements of the muscle contractile properties to gain insight into potential mechanisms of a walking exercise intervention on cancer-related fatigue. To ensure comprehensiveness, quantitative measurements were supplemented with cancer survivors’ experiences on how exercise helped to reduce fatigue severity. The current knowledge thereby provided useful leads to further improve exercise interventions aiming to reduce fatigue, such as the focus on improving muscle mass and function, and resilience.

A limitation of this study is the relatively small sample size to detect exercise intervention effects on fatigue. However, sample sizes of 27 are well accepted for studies using electrical stimulation [30, 40]. Due to the small sample size and the explorative nature of this study, our results should be interpreted as hypothesis-generating rather than hypothesis-testing. Additionally, we used a semi-interrupted time-series design instead of a randomized controlled trial in order to prevent unwillingness to participate when risking to be randomized into the control arm. Despite the strength of each person being its own control, it may have introduced contamination in the control period. This may have hampered the detection of significant changes on fatigue severity during the period of the walking intervention. Nevertheless, we were still able to study the factors longitudinally associated with fatigue severity. Another potential limitation of this study was the heterogeneity of the study population in terms of cancer type, treatment, and demographics. However, this may improve the generalizability to cancer survivors with cancer-related fatigue.

## Conclusion

The present study demonstrated that increases in muscle strength, physical activity, and sleep quality and decreases in muscle relaxation times and psychological distress were associated with reductions in fatigue severity in cancer survivors. Although these effects could not be directly attributed to the walking exercise intervention, our findings emphasize the importance of incorporating resistance and progressive muscle relaxation exercises aiming to improve muscle strength and muscle relaxation times and addressing important constructs such as resilience and physical well-being in a multimodal approach to improve the efficacy of interventions aimed at managing CRF.

## Supplementary Information

Below is the link to the electronic supplementary material.

Supplementary file1 (PDF 37 KB)

Supplementary file2 (PDF 217 KB)

Supplementary file3 (PDF 59 KB)

## Data Availability

The datasets generated during and/or analysed during the current study are available from the corresponding author on reasonable request.
