# Supplementary material for: Potential mechanisms underlying the effect of walking exercise on cancer-related fatigue in cancer survivors
Source: J Cancer Surviv. 2024 Jan 31;19(4):1132–42. doi: 10.1007/s11764-024-01537-y (PMC12283887; doi:10.1007/s11764-024-01537-y)
Supplement: Supplementary file 1 — Supplementary file1 (PDF 37 KB) [file 11764_2024_1537_MOESM1_ESM.pdf]

Supplementary material 1. *Behavioral change techniques applied during the walking intervention*

**Intervention phases**	**Applied behavioral change techniques (20)**	**Example of application in the KINETICS study**
Preparation	Information about health consequences	Researchers inform participants that scientific evidence indicates that exercise may help to reduce perceived fatigue.
		Researchers inform participants about the benefits of walking exercise and resistance exercise on fatigue.
	Goal setting	Researchers discuss with participants the final goal of this walking exercise program.
	Graded tasks	Researchers discuss with participants that the walking exercise program will be increased in small steps to heavier training sessions.
	Action planning	Researchers plan the exercise sessions together with the participant.
	Self-monitoring of behaviour	Participants identify factors that could hamper the planned behaviour. Participants self-monitor their behaviour during the intervention, which is discussed with the researcher.
	Problem solving	Researchers provide tools how to deal with factors hampering the planned behaviour.
	Teach to use prompts/cues	Researchers discuss with participants how they can turn the new behaviour into a routine
	Plan social support	Researchers discuss the possibility to ask for social support during the exercise sessions.
	Verbal persuasion to boost self-efficacy	Researchers keep in contact with the participant to make sure they set realistic goals.
	Instruction on how to perform the behaviour	Researchers provide the participant with clear instructions on how to perform the exercise sessions.
The exercise program*	Feedback on behaviour	Researchers ask the participants how the exercise sessions are going and give feedback on performance.
	Self-monitoring of behaviour	Participants monitor their behaviour for the past two weeks. Researchers discuss this behaviour with the participants.
	Problem solving	Researchers discuss with participants the encountered problems during the exercise sessions and potential solutions.
	Commitment	Researchers discuss with participants what is needed to complete the next exercise sessions.
	Action planning<br>Goal setting	Researchers discuss with participants whether the initial goal seems feasible, or if not what alternative goal participants would want to achieve.
	Focus on past success	Researchers emphasize previous achievements in the training program.
	Anticipation of future rewards	Researchers emphasize that participants are able to reach their final goal.

*Cancer survivors received counselling every other two weeks to monitor the training program and adjust the program if necessary
