# Supplementary material for: Potential mechanisms underlying the effect of walking exercise on cancer-related fatigue in cancer survivors
Source: J Cancer Surviv. 2024 Jan 31;19(4):1132–42. doi: 10.1007/s11764-024-01537-y (PMC12283887; doi:10.1007/s11764-024-01537-y)
Supplement: Supplementary file 2 — Supplementary file2 (PDF 217 KB) [file 11764_2024_1537_MOESM2_ESM.pdf]

***Supplementary material 2.*** *Descriptive information of potential factors underlying cancer-related fatigue.*

| | T0 (n = 27) | T1 (n = 24) | T2 ( n = 21) | Control period β (95% CI) | Intervention period β (95% CI) | Total study period β (95% CI) |
|---|---|---|---|---|---|---|
| **Physiological** | | | | | | |
| BMI ($kg/m^2$) | 26.9 ± 5.0 | 26.2 ± 3.5 | 25.9 ± 3.7 | 0.1 (-0.4 ; 0.6) | -0.3 (-0.8 ; 0.2) | -0.2 (-0.7 ; 0.3) |
| Skeletal muscle mass (kg) | 31.0 ± 5.0 | 31.3 ± 5.4 | 32.1 ± 4.8 | 0 (-0.4 ; 0.4) | -0.3 (-0.8 ; 0.1) | -0.3 (-0.7 ; 0.1) |
| Body fat mass (kg) | 24.4 ± 12.0 | 22.5 ± 8.5 | 21.4 ± 8.5 | 0.2 (-1.1 ; 1.4) | -0.6 (-1.9 ; 0.7) | -0.5 (-1.8 ; 0.8) |
| Estimated $VO_2max$ (ml/kg/min) | 27.5 ± 8.1 | 30.7 ± 11.5 | 34.0 ± 11.7 | 1.5 (-1.2 ; 4.3) | 3.3 (0.4 ; 6.3)* | 4.9 (2 ; 7.7)* |
| Estimated 1RM (kg) | 184.9 ± 57.9 | 219.2 ± 73.1 | 205.6 ± 69.3 | 28.3 (14.4 ; 42.3)* | -16.4 (-32.6 ; -0.2) | 11.9 (-3.1 ; 27.1) |
| Estimated 1RM (kg/kg body weight) | 2.3 ± 0.6 | 2.8 ± 0.9 | 2.6 ± 0.8 | 0.4 (0.2 ; 0.5)* | -0.2 (-0.4 ; 0) | 0.2 (0 ; 0.4) |
| MVC (N) | 484.9 ± 131.6 | 504.2 ± 140.3 | 504.9 ± 114.4 | 7.8 (-16.7 ; 32.5) | -25.3 (-51.4 ; 1.1) | -17.4 (-43.4 ; 8.9) |
| MVC (N/kg body weight) | 6.1 ± 1.8 | 6.4 ± 1.6 | 6.5 ± 1.5 | 0 (-0.3 ; 0.4) | -0.2 (-0.5 ; 0.2) | -0.1 (-0.5 ; 0.2) |
| Muscle fatiguability (%) | -29.3 ± 12.2 | -28.3 ± 12.8 | -25.9 ± 13.2 | 1.2 (-2.8 ; 5.2) | 2.5 (-1.9 ; 6.8) | 3.7 (-0.6 ; 7.9) |
| Early relaxation time (ms) | 24.0 ± 4.1 | 22.5 ± 3.1 | 24.5 ± 3.4 | -0.6 (-1.4 ; 0.3) | 1.5 (0.6 ; 2.4)* | 1 (0.1 ; 1.8)* |
| Increase early relaxation time (%) | 86.4 ± 43.0 | 90.3 ± 33.7 | 78.7 ± 41.5 | 4.1 (-8.9 ; 17) | -7.8 (-22 ; 6.2) | -3.7 (-17.5 ; 9.9) |
| Half relaxation time (ms) | 35.2 ± 7.2 | 32.6 ± 7.0 | 36.4 ± 4.4 | -1.8 (-5.4 ; 1.7) | 3.6 (-0.2 ; 7.4) | 1.8 (-2 ; 5.5) |
| Increase half relaxation time (ms) | 78.9 ± 48.2 | 90.5 ± 42.8 | 76.3 ± 47.7 | 11.6 (-7.8 ; 30.9) | -15.7 (-36.2 ; 5.2) | -4.1 (-24.5 ; 16.5) |
| Maximal force rise (%/ms) | 0.8 ± 0.2 | 0.7 ± 0.1 | 0.7 ± 0.3 | 0 (-0.1 ; 0) | 0 (-0.1 ; 0.1) | 0 (-0.1 ; 0) |
| Decrease Maximal force rise (%) | -5.6 ± 22.3 | -14.7 ± 11.9 | -19.2 ± 18.9 | -9.7 (-19.3 ; 0.1) | -3.9 (-14.4 ; 6.5) | -13.6 (-23.7 ; -3.4)* |
| SDNN | 25.0 (15.5 - 52.8) | 42.1 (27.8 - 70.8) | 23.7 (17.5 - 57.8) | 15.8 (-4.5 ; 35.7) | -21.8 (-43.2 ; -0.2) | -6 (-27.1 ; 14.9) |
| RMSSD | 26.3 (15.0 - 50.5) | 46.9 (20.4 - 77.6) | 28.8 (15.1 - 50.5) | 17.6 (-3.1 ; 37.9) | -21.7 (-43.5 ; 0.2) | -4.1 (-25.7 ; 17.3) |
| LF/HF ratio | 1.4 (0.7 - 4.4) | 1.8 (0.5 - 3.2) | 0.6 (0.4 - 1.8) | 1.9 (-0.9 ; 4.7) | -3.1 (-6.2 ; 0) | -1.2 (-4.2 ; 1.8) |
| TNF-α (pg/ml)† | 5.0 (0.1 - 12.0) | 5.0 (0.1 - 7.6) | 5.0 (5.0 - 6.0) | 1 (0.45 ; 2.2) | 2.7 (1.1 ; 6.7)* | 2.7 (1.1 ; 6.0)* |
| IL-6 (pg/ml)† | 0.5 (0.0 - 1.5) | 0.0 (0.0 - 1.0) | 0.0 (0.0 - 1.2) | 0.4 (0.2 ; 0.9)* | 1.6 (0.7 ; 4.1) | 0.7 (0.3 ; 1.5) |
| CRP (mg/l)† | 2.0 (0.0 - 2.8) | 0.0 (0.0 - 1.0) | 0.0 (0.0 - 1.5) | 0.7 (0.2 ; 2.2) | 0.9 (0.2; 3.3) | 0.6 (0.2 ; 2.2) |
| **Behavioral** | | | | | | |
| Daily step count | 8,812 (6,761 - 9,536) | 9,648 (7,643 - 10,899) | 10,921 (8,720 - 12,426) | 1069.8 (-137.6 ; 2293.9) | 1180.3 (-94 ; 2456.1) | 2250.1 (999.9 ; 3518.2)* |
| MVPA (hours/week) | 7.6 ± 2.9 | 8.8 ± 3.7 | 10.1 ± 3.3 | 1 (-0.2 ; 2.2) | 1.2 (0 ; 2.5) | 2.2 (1 ; 3.5)* |
| Sitting time (hours/day) | 10 ± 2 | 9 ± 1 | 9 ± 1 | 0 (-0.5 ; 0.6) | -0.3 (-0.9 ; 0.3) | -0.3 (-0.8 ; 0.3) |
| Self-reported MVPA leisure time (hours/week) | 7.1 (3.7 - 11.5) | 4.8 (1.9 - 10.9) | 7.5 (3.1 - 14.6) | -2 (-5.5 ; 1.6) | 2.5 (-1.3 ; 6.2) | 0.5 (-3.2 ; 4.2) |
| Self-reported MET-hours/week† | 92.1 (61.4 - 132.9) | 75.0 (62.2 - 107.3) | 105.2 (60.0 - 211.4) | 0.9 (0.7 ; 1.2) | 1.2 (1 ; 1.6) | 1.1 (0.9 ; 1.5) |
| Global sleep quality†† | 7 ± 3 | 7 ± 4 | 6 ± 3 | -0.1 (-1 ; 0.9) | -0.6 (-1.7 ; 0.4) | -0.7 (-1.7 ; 0.3) |
| **Psychological** | | | | | | |
| Distress† | 7 (4 – 12) | 7 (4 – 10) | 8 (3 – 10) | -0.9 (-2.6 ; 0.8) | 0.3 (-1.5 ; 2.1) | -0.6 (-2.4 ; 1.2) |

***Appendix II.*** *Values are presented as mean ± SD for normally distributed values or median (IQR) for not normally distributed value. Regression coefficients (β) and 95% confidence intervals (CI) represent the mean change in the variable over time assessed using unadjusted linear mixed models. †β (95% CI) of these variables were log-transformed and are presented in this column after back transformation to original scale. ††Higher scores represent lower sleep quality and distress. Abbreviations: BMI; body mass index, $VO_2max$; maximum oxygen uptake, 1RM; 1 repetition maximum; SDNN, standard deviation of NN intervals, RMSSD; root mean square of successive RR interval differences, LF/HF ratio; low frequency/high frequency ratio, MVPA; moderate to vigorous physical activity, MET; metabolic equivalent of task.*
** = p-value < 0.05*
