# Supplementary material for: Potential mechanisms underlying the effect of walking exercise on cancer-related fatigue in cancer survivors
Source: J Cancer Surviv. 2024 Jan 31;19(4):1132–42. doi: 10.1007/s11764-024-01537-y (PMC12283887; doi:10.1007/s11764-024-01537-y)
Supplement: Supplementary file 3 — Supplementary file3 (PDF 59 KB) [file 11764_2024_1537_MOESM3_ESM.pdf]

Supplementary material 3*. Individual statements with mean importance used for concept mapping analysis*

Focus statement: *The walking exercise influenced my perceived fatigue because:*

**Statement name**		**Importance ± SD**
**Resilience**		**3.89 ± 0.28**
11	I am more positive in life	4.00 ± 0.69
35	Over time, I was able to physically recover faster after the exercise training	3.67 ± 0.69
43	I was able to do more (more kilometres) than I expected	3.67 ± 0.77
44	I was surprised by the resilience of my body	3.78 ± 0.81
46	People around me noticed changes in my body	4.33 ± 0.59
**Physical well-being**		**3.88 ± 0.46**
3	I now have a reason for feeling fatigued	2.89 ± 1.23
7	I am more active	4.56 ± 0.51
13	I enjoyed going to places I had never been before/discovering new routes	4.22 ± 0.73
23	I gained more endurance/fitness	4.33 ± 0.59
30	I noticed that my boundaries were being pushed	3.61 ± 1.04
34	I recover faster from fatigue	3.33 ± 0.84
39	I changed my eating habits (more/larger portions)	4.11 ± 0.58
42	It promotes my gut recovery	3.78 ± 0.65
45	I felt that I looked better	4.11 ± 0.83
47	People around me noticed changes in my appearance	3.72 ± 0.67
48	I feel healthy	4.11 ± 0.47
60	I sleep better	3.78 ± 0.65
**Daily functioning**		**3.77 ± 0.48**
5	I can walk hills and bridges more easily	3.94 ± 0.73
10	I felt happier	4.06 ± 0.80
17	I adapted my daily routine to the exercise schedule, and I liked that	3.89 ± 0.90
20	I felt more energy after the exercise sessions	3.11 ± 1.02
22	I felt better after a walking exercise session	4.39 ± 0.61
24	It was nice to walk together	3.11 ± 1.32
27	I learned to spread my energy throughout the day	3.89 ± 0.96
**Physical fitness**		**3.63 ± 0.50**
6	I feel physically fitter	4.22 ± 0.55
9	I have become stronger	3.72 ± 0.75
12	I noticed that I liked the exercise structure	3.17 ± 0.99
18	I noticed less mental fatigue	4.00 ± 0.59
29	I have discovered that there is a limit to what is most enjoyable when it comes to walking	3.78 ± 0.43
36	The walking training gave me the opportunity to take time for myself	4.06 ± 1.00
38	I have more interest in doing/room for fun things	2.72 ± 1.07
51	I noticed that I had to take the fatigue into account during the exercise sessions	4.11 ± 0.58
52	I used the walking training to stay as fit as possible	4.22 ± 0.65
53	I found the strength training a pleasant variation because it targets different muscles	4.06 ± 0.64
55	The moments when I was completely overwhelmed by fatigue disappeared	3.28 ± 1.23
57	I noticed that it took longer for the fatigue to set in	3.17 ± 1.10
59	I quickly notice muscle fatigue which can be alleviated by walking	3.22 ± 1.00
61	I have more leg muscles and less fat mass	3.11 ± 0.83
**Training benefits**		**3.12 ± 0.40**
1	I felt less fatigued by the training sessions	3.39 ± 0.85
16	I liked the challenge	3.39 ± 1.20
21	Walking helps me to worry less	3.33 ± 1.19
58	I used the training to improve my walking technique	2.72 ± 1.13
62	I found it important that someone provided guidance during the exercise training	2.50 ± 1.29
63	I appreciated that the training was easily accessible	3.39 ± 1.09
**Health awareness**		**3.00 ± 0.34**
2	I have a better insight into the fatigue	3.06 ± 1.30
14	It felt good to be outside	3.17 ± 1.04
15	I found it relaxing to be outside	3.33 ± 1.14
25	I make different social contacts through walking than I normally would	3.39 ± 0.92
26	I need less planning to have more energy	3.17 ± 1.04

31	I noticed new or positive things during walking	3.33 ± 0.84
32	Each time, the training sessions went better and better	2.56 ± 0.70
40	I am more aware of my dietary intake	2.28 ± 1.02
41	I can go longer without eating	3.11 ± 0.90
49	I liked being engaged with my health in a positive way	2.72 ± 1.07
54	I noticed positive effects on other exercises too	2.78 ± 1.00
56	Fatigue disappears while walking	3.11 ± 1.13
**Mental well-being**		**2.95 ± 0.46**
4	Mental fatigue turned into physical fatigue	3.33 ± 0.91
8	I can do more (daily) activities	3.56 ± 0.98
19	I noticed positive mental effects	2.72 ± 1.07
28	I get happy from walking	2.78 ± 1.17
33	I noticed that I started to walk quicker	2.89 ± 1.28
37	I noticed that I felt comfortable in my own skin	2.17 ± 0.92
50	I felt proud after a long walk.	3.22 ± 0.94
